# With changes to NHS England commissioning policies for drugs used in rheumatology, is the role of the specialist commissioning multidisciplinary team obsolete?

**DOI:** 10.1093/rap/rkag139

**Published:** 2026-10-30

**Authors:** Zoe Syrimi, Zoe McLaren, Robert J Moots, Devesh Mewar, Rosalind Benson

**Affiliations:** Department of Rheumatology, NHS University Hospitals of Liverpool Group, Liverpool, United Kingdom; Department of Rheumatology, NHS University Hospitals of Liverpool Group, Liverpool, United Kingdom; Department of Rheumatology, NHS University Hospitals of Liverpool Group, Liverpool, United Kingdom; Department of Rheumatology, NHS University Hospitals of Liverpool Group, Liverpool, United Kingdom; Department of Rheumatology, NHS University Hospitals of Liverpool Group, Liverpool, United Kingdom

Key message

- Regional specialist commissioning MDTs provide clinical value beyond funding decisions.

Dear Editor, Since April 2026, several drugs used in rheumatology are no longer classified as high-cost drugs (HCDs) under NHS England (NHSE) commissioning arrangements [1]. These include rituximab for refractory SLE, idiopathic inflammatory myopathies (IIMs), ANCA-associated vasculitis (AAV) and IgG4-related disease and infliximab for refractory sarcoidosis. Consequently, NHSE no longer requires specialist commissioning multidisciplinary team (MDT) approval to fund these treatments. These changes prompted us to evaluate the ongoing role and value of the Cheshire & Mersey specialist commissioning MDT.

The MDT was established at NHS University Hospitals of Liverpool Group (UHLG) in 2022 and meets online monthly, covering a population of approximately 2.7 million. We conducted a retrospective service evaluation of all referrals to the MDT over a 12-month period. Approval for the service evaluation was provided by the Trust Audit Teams at UHLG. Ethical approval was not required as it met criteria for service evaluation. Audit code: Rheum/SE/2026–27/03.

Seventy-seven patients were referred, of whom 76 were discussed. Forty-six discussions were for treatment approval, and 30 were presented for noting following treatment approval by MDT members outside the meeting. Forty-six patients were from UHLG, 25 from five local acute trusts (non-specialist commissioning centres) and five received shared care between UHLG and a local trust. Treatment requests and their indications are summarised in Table 1. Two patients were excluded from the analysis because MDT approval was no longer required. Overall, 70 treatment requests (95%) were approved, including three cases in which one of two proposed treatment requests was recommended. In two cases, additional treatments were recommended alongside the approved drug and in one case two options were given. Twenty requests from local acute trusts (87%) were approved. For unapproved requests, management advice was given in all cases and an alternative HCD was recommended for three patients. Of the 76 cases discussed, 49 (64%) were for drugs that are now in tariff.

**Table 1:** Drug requests and their indications discussed at the Cheshire and Mersey specialist commissioning MDT.

Disease	Drug requested	Number of patients (*n*)
Refractory SLE	Rituximab	19
	Belimumab	8
	Obinutuzumab or belimumab	1
Refractory IIMs	Rituximab	18
	Abatacept	1
	Rituximab or abatacept	1
AAV	Rituximab	13
	Benralizumab	2
Refractory digital ulcers	Bosentan	4
Refractory AOSD	Tocilizumab	2
	Anakinra	1
GCA	Tocilizumab extension or retreatment	2
IgG4-RD	Rituximab	2
Refractory Takayasu arteritis	Tocilizumab	1
Refractory sarcoidosis	Infliximab	1

IIMs: idiopathic inflammatory myopathies; AAV: ANCA-associated vasculitis; AOSD: adult-onset Still’s disease; IgG4-RD: IgG4-related disease.

Beyond funding decisions, the MDT provided additional recommendations for 30 patients (39.5%). These included advice on current treatment (*n* = 16), future management (*n* = 15), investigations (*n* = 6), clinical trials (*n* = 3), involvement of other specialties (*n* = 2), pregnancy planning (*n* = 1) and support for local funding applications (*n* = 3). Importantly, the MDT made additional recommendations for 20 patients (80%) from local acute trusts. Among UHLG cases presented for noting, additional recommendations were made for five patients (17%).

Regional specialised rheumatology networks began in 2015–16 aiming to link all specialised and non-specialised rheumatology units in the 12 regions of England and ensure equitable access and uniform standards of care [2]. From 2016 to 2019, NHSE offered a CQUIN to support the development of regional MDTs for patients with multisystem autoimmune rheumatic diseases, recognising variation in standards of care and outcomes depending on where patients were treated [3]. The Rheumatology GIRFT National Specialty Report (2021) subsequently made recommendations to improve equitable access to specialised care through regional networks. The report highlighted that, in some trusts, no formal mechanism exists for discussing cases at virtual MDT meetings, which can delay initiation of HCD treatments [2]. Although specialist MDT care has been associated with improved clinical outcomes in AAV [4], evidence remains limited on the impact of regional specialist commissioning MDTs on patient outcomes. Recent studies of regional HCD MDT models in nephrology and dermatology have described benefits of their services including access to expert clinical advice, support for HCD access, improved adherence to guidelines, access to clinical trials and registries and education [5, 6]. The 2025 British Society for Rheumatology management recommendations for AAV continue to recommend that specialist centres provide regional MDTs, including mechanisms to approve HCDs [7].

Specialist commissioning MDTs are resource-intensive, requiring time and expertise from multiple clinicians to review cases and attend meetings. As NHSE commissioning requirements change, this raises an important question about whether the resources required to maintain this service remain justified. Our evaluation demonstrates that the MDT provides clinical value beyond funding decisions, with additional recommendations in almost 40% of cases overall and 80% of referrals from local acute trusts. This suggests that the MDT’s advisory function may be particularly relevant to non-specialist centres.

A previous evaluation of patient experiences of accessing out-of-tariff medications in our region demonstrated a preference for local treatment provision, minimising the need to travel to specialist centres. However, patients managed through shared-care arrangements valued the specialist expertise provided by our centre alongside local care delivery. A previous regional survey of clinicians from local acute trusts in our region demonstrated that they were keen to deliver out-of-tariff drugs locally after approval and with support from our specialist centre. It will be important to re-evaluate referring clinicians’ views following the changes in NHSE commissioning policies.

Funding approval by our MDT remains required by NHSE for belimumab for refractory SLE, abatacept for refractory IIMs and severe treatment-resistant morphoea, anakinra for haemophagocytic lymphohistiocytosis, bosentan for refractory digital ulceration in systemic sclerosis and tocilizumab for Takayasu arteritis and refractory adult-onset Still’s disease. As new HCDs enter clinical practice, specialist commissioning MDTs will likely remain required to make funding approval decisions.

As more treatments move in tariff, the role of specialist commissioning MDTs may evolve from funding gatekeepers to a regional expert advisory network. Such a model could provide a platform for non-specialist centres to access specialist multidisciplinary expertise, supporting local provision of care and serving as a forum for discussing complex cases.

## Data Availability

The data underlying this article will be shared on reasonable request to the corresponding author.
